# Epigenetic Histone Methylation of PPARγ and CPT1A Signaling Contributes to Betahistine Preventing Olanzapine-Induced Dyslipidemia

**DOI:** 10.3390/ijms24119143

**Published:** 2023-05-23

**Authors:** Yueqing Su, Chao Deng, Xuemei Liu, Jiamei Lian

**Affiliations:** 1 Fujian Maternity and Child Health Hospital, College of Clinical Medicine for Obstetrics & Gynaecology and Paediatrics, Fujian Medical University, Fuzhou 350005, China; syq0506@126.com; 2 Antipsychotic Research Laboratory, Illawarra Health and Medical Research Institute, Wollongong, NSW 2522, Australia; chao@uow.edu.au; 3 School of Medical, Indigenous and Health Sciences, and Molecular Horizons, University of Wollongong, Wollongong, NSW 2522, Australia; 4 School of Pharmaceutical Sciences, Medical Research Institute, Southwest University, Chongqing 400716, China; liuxm@swu.edu.au

**Keywords:** betahistine, olanzapine, dyslipidemia, PPARγ, CPT1A, histone methylation

## Abstract

As a partial histamine H1 receptor agonist and H3 antagonist, betahistine has been reported to partially prevent olanzapine-induced dyslipidemia and obesity through a combination therapy, although the underlying epigenetic mechanisms are still not known. Recent studies have revealed that histone regulation of key genes for lipogenesis and adipogenesis in the liver is one of the crucial mechanisms for olanzapine-induced metabolic disorders. This study investigated the role of epigenetic histone regulation in betahistine co-treatment preventing dyslipidemia and fatty liver caused by chronic olanzapine treatment in a rat model. In addition to abnormal lipid metabolism, the upregulation of peroxisome proliferator-activated receptor γ (PPARγ) and CCAAT/enhancer binding protein (C/EBPα), as well as the downregulation of carnitine palmitoyltransferase 1A (CPT1A) in the liver induced by olanzapine, were significantly attenuated by betahistine co-treatment. In addition, betahistine co-treatment significantly enhanced the global expression of H3K4me and the enrichment of H3K4me binding on the promoter of *Cpt1a* gene as revealed by ChIP-qPCR, but inhibited the expression of one of its site-specific demethylases, lysine (K)-specific demethylase 1A (KDM1A). Betahistine co-treatment also significantly enhanced the global expression of H3K9me and the enrichment of H3K9me binding on the promoter of the *Pparg* gene, but inhibited the expression of two of its site-specific demethylases, lysine demethylase 4B (KDM4B) and PHD finger protein 2 (PHF2). These results suggest that betahistine attenuates abnormal adipogenesis and lipogenesis triggered by olanzapine through modulating hepatic histone methylation, and thus inhibiting the PPARγ pathway-mediated lipid storage, while at the same time promoting CP1A-mediated fatty acid oxidation.

## 1. Introduction

Second-generation antipsychotics (SGAs), such as olanzapine, have a remarkable therapeutic effect in schizophrenia and other psychotic disorders [1]. However, SGA treatment induces obesity and other severe metabolic disorders [2,3]. Accumulated evidence has revealed that multiple neurotransmitter receptors, including the histaminergic H1, muscarinic M3, and serotonin 5-HT2C receptors, contribute to SGA-induced weight gain/obesity and other metabolic side effects. Of these, the H1 receptor has been identified as a main indicator predicting weight gain induced by SGAs [4,5,6,7]. Betahistine acts as a modulator of the histaminergic system and has both H1 receptor agonistic and H3 receptor antagonistic properties in the brain [8,9]. Although the preclinical results remain to be completely replicated by clinical studies, recent evidence has demonstrated that olanzapine-induced weight gain and metabolic side effects were significantly alleviated by co-treatment with betahistine in both preclinical animal models and clinical trials [4,10,11,12,13,14]. However, the underlying mechanisms have still not been well investigated. Using a rat model, it has been revealed that betahistine may reduce/prevent olanzapine-induced weight gain, partially through modulating the hypothalamic histamine H1 receptor–AMP activated protein kinase (AMPK)–neuropeptide Y (NPY) pathway [4,15]. Recent studies have reported that peripheral metabolic organs such as the liver participate in the modulation of olanzapine-induced dyslipidemia side effects, while betahistine could alleviate dyslipidemia and fatty liver caused by olanzapine treatment [16,17].

Peroxisome proliferator-activated receptor gamma (PPARγ), as one of three PPARs (PPAR-α, PPARδ/β, and PPAR-γ), is mostly involved in the regulation of adipogenesis, the energy balance, and lipid biosynthesis [18,19]. Evidence from both animal and clinical studies has revealed that modification of the PPARγ regulatory system is a therapeutic target for hepatic diseases and lipid disorders [20,21,22,23]. Our recent study in a rat model observed that hepatic dyslipidemia/adipogenesis caused by olanzapine was correlated with histone modulation of the PPARγ pathway and lipid storage [24]. Carnitine palmitoyltransferase 1A (CPT1A), as one of the three isoforms of CPT1, is mainly expressed in the liver, and its master role has been well recognized in triglyceride metabolism [25]. Moreover, CPT1A is a key enzyme function for fatty acid oxidation in mitochondria of the liver through modulating fatty acid uptake in the mitochondria [26]. CPT1A gene polymorphisms were associated with metabolic disturbance in woman during pregnancy [27]. Our previous study showed that the hepatic SREBP–CPT1A pathway exhibited downregulation after chronic olanzapine treatment, while it was upregulated in both betahistine-only and betahistine co-treatment rats [16]. Accumulated evidence in recent years has demonstrated that epigenetic mechanisms play critical roles in the transcriptional regulation of *Cpt1a*, including DNA methylation, miRNAs, and histone modification [26,28,29]. However, it is still unknown whether epigenetic modulation is a mechanism involved in the therapeutic effect of betahistine on olanzapine-induced dyslipidemia, which has been addressed in this study.

It is well known that chromatin can repress or activate mRNA transcription through dynamically altering their structures [30]. Modification at the special site of lysine (K) residues on the histone N-terminal tail affects the chromatin structure and gene expression [31]. Generally, the methylation of K4 on histone 3 (H3K4me) is linked to gene activation, while the methylation of K9 or methylation of K27 on H3 (H3K9me or H3K27me) represses gene expression [32]. It has been reported that H3K4me, H3K9me, and H3K27me are associated with the expression of CPT1A, PPARγ, and C/EBPα [24,33,34,35]. In addition, it is well known that histone methylations are dynamically written by site- and cell-specific histone methyltransferases and erased by histone demethylases [36]. Therefore, this study measured the global changes in these histone marks and their enrichment binding on the promoter regions of the above genes in female rats after treatment with olanzapine and/or betahistine. In addition, the expression of the list of position- and cell-type-specific histone methyltransferases (such as KMT2C, EHMT2, and EZH2) and demethylases (such as KDM1A, KDM4B, and PHF2) was also examined.

## 2. Results

### 2.1. Betahistine Co-Treatment Ameliorated Dyslipidemia Caused by Olanzapine

**Figure 1:**
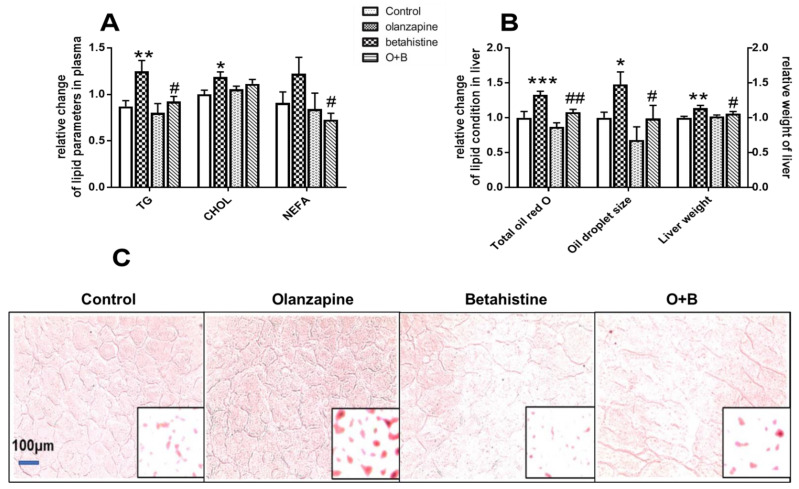
The effects of chronic olanzapine and/or betahistine treatment on (**A**) plasma triglycerides (TG) and non-esterified fatty acid (NEFA). (**B**) Data from Oil Red O (ORO) staining of liver sections are presented. (**C**) Image of hepatic ORO staining. Data are presented as mean ± SEM (*n* = 12 per group). * *p* < 0.05, ** *p* < 0.01, *** *p* < 0.001, vs. control; # *p* < 0.05, ## *p* < 0.01, vs. olanzapine. O+B, co-treatment of olanzapine and betahistine.

As in our previous report, betahistine co-treatment significantly reversed the increased food intake and weight gain induced by olanzapine treatment [13]. As shown in Figure 1A, the rats with olanzapine-only treatment had significantly higher plasma TG and TC levels than the control (*p* < 0.01, *p* < 0.05, respectively). However, only a significant decrease in TG, but not TC levels, was observed in the O+B co-treatment group compared to the olanzapine-only group (*p* < 0.05). Meanwhile, although only a slight increase in the plasma NEFA concentration was observed in the olanzapine-only group compared to the control (*p* > 0.05), the NEFA level was significantly lower in the O+B group than in the olanzapine-only group (*p* < 0.05).

As shown in Figure 1B, olanzapine treatment led to a significant increase in liver weight compared to the control (*p* < 0.05), while O+B co-treatment significantly reduced the liver weight compared to the olanzapine-only group (*p* < 0.05). Figure 1C presents representative images of Oil-Red-O-stained histological sections of livers. Olanzapine treatment significantly increased the total Oil Red O (*p* < 0.001) and the size of lipid drops (*p* < 0.01) in the hepatic tissue compared to the control, while the increases were significantly reversed by O+B co-treatment (vs. olanzapine-only, *p* < 0.01 and *p* < 0.05, respectively; Figure 1B,C).

### 2.2. Betahistine Co-Treatment Partly Alleviated Activation of the PPARγ/C/EBPα Pathway Induced by Olanzapine

**Figure 2:**
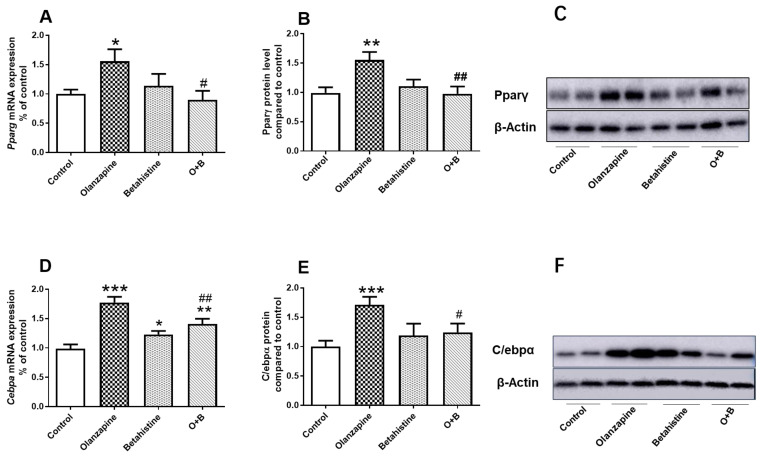
Effect of chronic olanzapine and/or betahistine treatment on PPARγ pathway. (**A**) mRNA expression of *Pparg*; (**B**) relative protein levels of Pparγ; (**C**) representative images of Western blot for PPARγ (57-KDa) and β-actin (42-KDa; as loading controls); (**D**) mRNA expression of *Cebpa*; (**E**) relative protein levels of C/ebpα; (**F**) representative images of Western blot for C/ebpα (43-KDa) and β-actin (42-KDa; as loading controls). Data are presented as mean ± SEM. The sample size is 6 per group. * *p* < 0.05, ** *p* < 0.01, *** *p* < 0.001, vs. control; # *p* < 0.05, ## *p* < 0.01, vs. olanzapine. Abbreviations: C, control; O, olanzapine; B, betahistine; O+B, co-treatment of olanzapine and betahistine.

For PPARγ, there was a significant interaction between olanzapine and betahistine on both its mRNA expression (F_1,18_ = 5.307, *p* = 0.033) and protein level (F_1,20_ = 8.41, *p* = 0.009). The *Pparg* mRNA level in the olanzapine-only group was significantly increased compared to the control (*p* < 0.05), while it was significantly decreased by O+B co-treatment (vs. olanzapine-only, *p* < 0.05) (Figure 2A). Similarly, the Pparγ protein concentration was significantly higher in olanzapine-only rats (vs. control, *p* < 0.01), while it was significantly lower in the O+B group (vs. olanzapine, *p* < 0.01) (Figure 2B,C).

For C/EBPα, there was a significant main effect of the olanzapine factor (F_1,20_ = 36.460, *p* < 0.001) and a significant interaction between olanzapine and betahistine (F_1,20_ = 14.360, *p* = 0.001) on mRNA expression. Further post-hoc analysis showed the significant upregulation of *Cebpα* mRNA in the olanzapine-only-treated group compared to the control group (*p* < 0.001; Figure 2D). Although a significant increase in *Cebpα* mRNA was observed in the betahistine-only group (vs. control, *p* < 0.05), co-treatment with betahistine partially but significantly reduced *Cebpα* mRNA expression (O+B vs. olanzapine-only, *p* < 0.01; O+B vs. Control, *p* < 0.01; Figure 2D). Consistently, there was a significant main effect of the olanzapine factor (F_1,19_ = 6.679, *p* = 0.018) and an interaction between these two factors (F_1, 19_ = 4.937, *p* = 0.039) on C/ebpα protein levels. A post-hoc analysis also showed that the C/EBPα protein levels in the olanzapine-only group were significantly higher than those of the control (*p* < 0.001), while O+B co-treatment significantly reduced the C/ebpα protein levels (vs. olanzapine-only, *p* < 0.05; Figure 2E,F). Additionally, a strong positive correlation between Pparγ and C/ebpα protein levels was observed (*r* = 0.402, *p* = 0.032).

### 2.3. Betahistine Co-Treatment Reversed the CPT1A Inhibition Induced by Olanzapine

**Figure 3:**
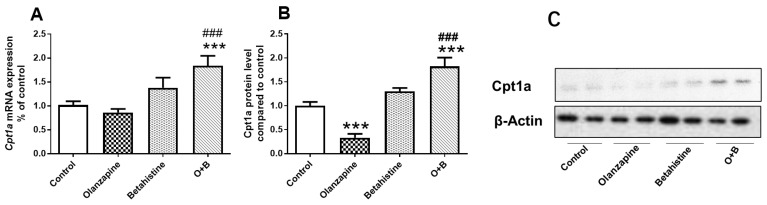
Effect of chronic olanzapine and/or betahistine treatment on CPT1A expression. (**A**) mRNA expression of *Cpt1a*; (**B**) relative protein levels of *Cpt1a*; (**C**) representative images of Western blot for Cpt1a (87-KDa) and β-actin (42-KDa; as loading controls). Data are presented as mean ± SEM (*n* = 6/group). *** *p* < 0.001, vs. control; ### *p* < 0.001, vs. olanzapine. Abbreviations: C, control; O, olanzapine; B, betahistine; O+B, co-treatment of olanzapine and betahistine.

This study observed a main effect of the betahistine factor (F_1,20_ = 18.16, *p* < 0.001) and a trend of interaction between olanzapine and betahistine on *Cpt1a* mRNA levels (F_1,20_ = 3.847, *p* = 0.063). There was also a main effect of the betahistine factor (F_1,20_ = 61.640, *p* < 0.001) and a significant interaction between these two drugs (F_1,20_ = 27.900, *p* < 0.001) on the *Cpt1a* protein concentration. Although olanzapine-only treatment did not significantly decrease *Cpt1a* mRNA expression (Figure 3A), it significantly decreased the *Cpt1a* protein levels compared to the control (*p* < 0.001; Figure 3B). The betahistine-only treatment tended to increase *Cpt1a* mRNA expression (*p* = 0.064) and *Cpt1a* protein levels (*p* = 0.075) compared to the control. The O+B co-treatment significantly upregulated *Cpt1a* mRNA (vs. control, *p* < 0.001; vs. olanzapine-only, *p<* 0.001) and reversed the olanzapine-induced decrease in *Cpt1a* protein levels (O+B vs. control, *p<* 0.001; O+B vs. olanzapine-only, *p<* 0.001; Figure 3).

### 2.4. Global Profile of H3K4me, H3K9me, and H3K27me in the Hepatic Tissue

**Figure 4:**
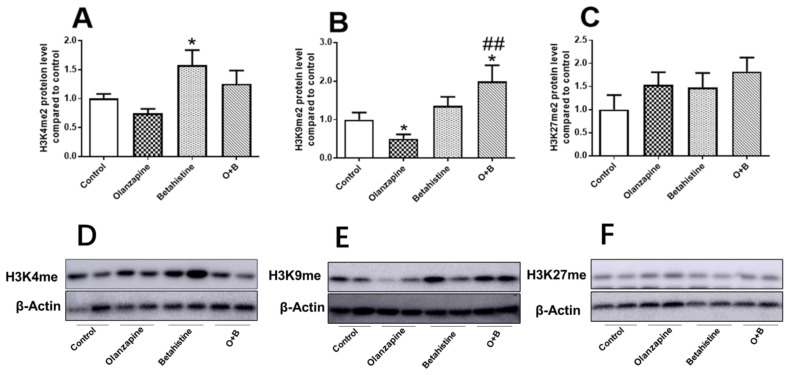
Effect of chronic olanzapine and/or betahistine treatment on global histone methylation. Protein levels of (**A**) H3K4me2, (**B**) H3K9me2, (**C**) H3K27me2; representative images of Western blot (β-actin 42-KDa acted as control) for (**D**) H3K4me2 (15 KDa), (**E**) H3K9me2 (17 KDa), (**F**) H3K27me2 (17 KDa). Data are presented as mean ± SEM (*n* = 6/group); * *p* < 0.05, vs. control; ## *p* < 0.01, vs. olanzapine. Abbreviations: C, control; O, olanzapine; B, betahistine; O+B, co-treatment of olanzapine and betahistine.

As shown in Figure 4A,D, in the case of global H3K4me2 levels, betahistine-only treatment significantly upregulated global H3K4me2 expression (vs. control, *p* < 0.05), while O+B showed a trend of increased global H3K4me2 compared to the olanzapine-only group (*p =* 0.064) (Figure 4A). The global H3K9me2 level was significantly decreased in olanzapine-only-treated rats (vs. control, *p* < 0.05), while it was significantly increased in the O+B co-treatment rats (vs. control, *p* < 0.05; vs. olanzapine-only, *p* < 0.01) (Figure 4B,E). There were not any significant differences between treatments in the global H3K27me2 protein level (all *p* > 0.05, Figure 4C,F).

### 2.5. Betahistine Co-Treatment Reversed the Activation of Kdm1a and Kdm4 Induced by Olanzapine

To further explore the role of site-specific enzymes in H3K4me, H3K9me, and H3K27me modification, the mRNA levels of three histone methyltransferases and three demethylases were analyzed using qPCR. They were KMT2C (methyltransferase for H3K4me), EHMT2 (methyltransferase for H3K9me), EZH2 (methyltransferase for H3K27me), KDM1A (demethylase for both H3K4me and H3K9me), KDM4B (demethylase for H3K9me), and PHF2 (demethylase for both H3K9me and H3K27me).

**Figure 5:**
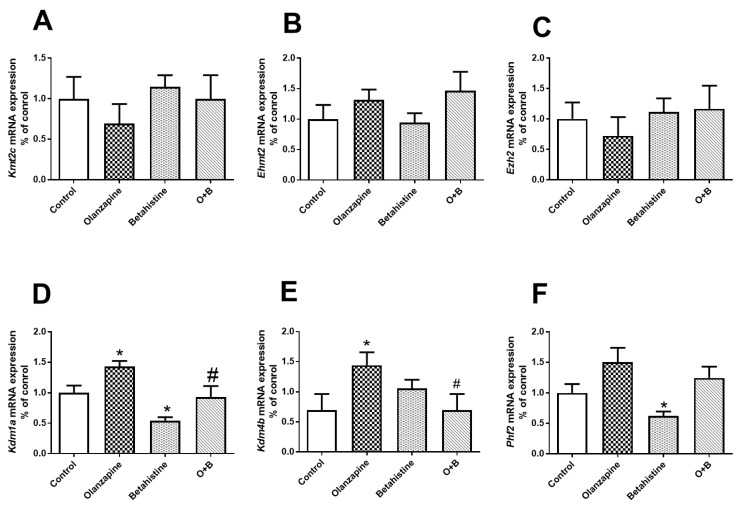
Effects of chronic olanzapine and/or betahistine treatment on mRNA expression of histone site-specific enzymes. Histone methyltransferases (HMTs) of (**A**) Kmt2c (H3K4me), (**B**) Ehmt2 (H3K9me), (**C**) Ezh2 (H3K27me); histone demethylase (HDMs) of (**D**) Kdm1a (H3K4me and H3K9me), (**E**) Kdm4b (H3K9me), (**F**) Phf2 (H3K9me and H3K27me). Data are presented as mean ± SEM (*n* = 6/group); * *p* < 0.05, vs. control; # *p* < 0.05, vs. olanzapine. O+B, co-treatment of olanzapine and betahistine.

For *Kdm1a* mRNA expression, there were significant main effects of the olanzapine (F_1,18_ = 9.646, *p* = 0.006) and betahistine factors (F_1,18_ = 13.330, *p* = 0.002). Compared to the control, the *Kdm1a* mRNA level was significantly higher in olanzapine-only-treated rats (*p* < 0.05), but lower in the betahistine-only group (*p* < 0.05). Moreover, co-treatment with betahistine significantly reduced *Kdm1a* mRNA expression, elevated by olanzapine (olanzapine-only vs. O+B co-treatment, *p* < 0.05) (Figure 5D). There was a significant interaction between the olanzapine and betahistine factors on *Kdm4b* mRNA expression (F_1,20_ = 5.814, *p* = 0.026). *Kdm4b* levels significantly increased in olanzapine-only rats (vs. control, *p* < 0.05), but were significantly decreased in O+B co-treatment rats (vs. olanzapine-only, *p* < 0.05; Figure 5E). In comparison with the control, there was higher expression of *Phf2* mRNA in the olanzapine-only rats (*p* = 0.051), but significantly lower Phf2 mRNA in the betahistine-only rats (*p* < 0.05). However, there were no significant changes in the mRNA expression of *Kmt2c*, *Ehmt2*, or *Ezh2* (all *p* > 0.05, Figure 5A–C).

### 2.6. Betahistine Co-Treatment Increased the Enrichment of H3k9me3 Binding on the Promoter Region Pparg2, and H3K4me2 Binding on the Promoter Region Cpt1a

**Figure 6:**
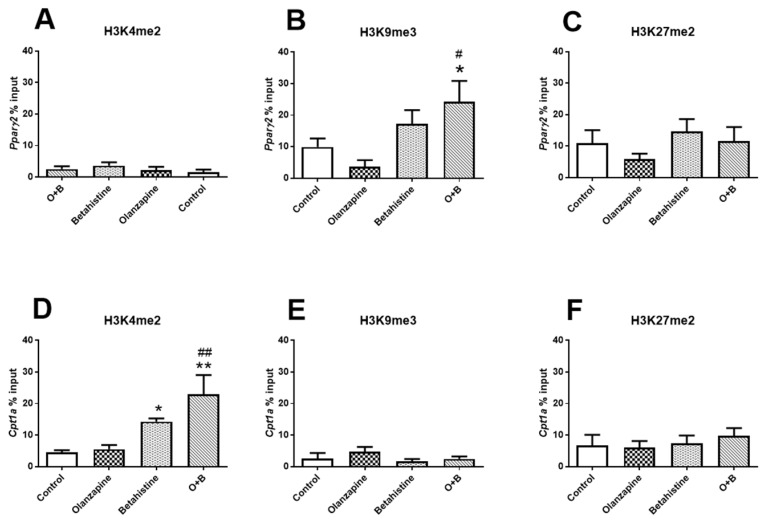
Histone methylation on promoters of *Pparg2* and *Cpt1a*. Histone modifications on *Pparg2* (**A**–**C**) and *Cpt1a* (**D**–**F**) genes were measured by ChIP-qPCR with antibodies against (**A**,**D**) H3k4me2 (active mark), (**B**,**E**) H3K9me3 (inactive mark), and (**C**,**F**) H3K27me2 (inactive mark) in the livers of rats treated with olanzapine and/or olanzapine control (*n* = 6/group). Data are presented as mean ± SEM. * *p* < 0.05, ** *p* < 0.01, vs. control; # *p* < 0.05, ## *p* < 0.01, vs. olanzapine. O+B, co-treatment of olanzapine and betahistine.

As revealed by the ChIP-qPCR experiment (Figure 6B), the amount of H3K9me3 binding peaking on the promoter region of *Pparg2* tended to be decreased in the olanzapine-only rats (vs. control, *p* = 0.082), while it was significantly increased in those with O+B co-treatment (both *p* < 0.05). No significant changes in H3K4me2 or H3K27me2 binding were observed on the promoter region of *Pparg2* (all *p* > 0.05, Figure 6A,C). On the other hand, H3K4me2 significantly increased binding on the promoter region of *Cpt1a* in both the betahistine-only group and O+B group (vs. control, *p* < 0.05 and *p* < 0.01, respectively; Figure 6D). Although there was no significant difference between the olanzapine-only and the control groups (*p* > 0.05), H3K4me2 peaking on the promoter region of *Cpt1a* was significantly higher in the O+B co-treated rats than olanzapine-only rats (*p* < 0.01; Figure 6D). There were no changes in H3K9me3 or H3K27me2 binding on the promoter region of *Cpt1a* (all *p* > 0.05, Figure 6E,F). Neither H3K4me2 nor H3K9me3 were detected on the promoter regions on *Pparg1*.

## 3. Discussion

This study explored the epigenetic histone methylation mechanisms in the amelioration effect of betahistine co-treatment on dyslipidemia after chronic olanzapine administration in a female rat model. As reported previously [15,16], the hyperlipidemia and hepatic lipid/adipocyte accumulation caused by chronic olanzapine treatment were ameliorated by four weeks of co-treatment of betahistine and olanzapine. The upregulation of hepatic Pparγ and C/ebpα (master regulators in adipogenesis) induced by olanzapine was reversed by co-treatment with betahistine. On the other hand, as reported previously [16], the expression of *Cpt1a* (the limiting enzyme related to the progress of fatty acid oxidation) was downregulated by olanzapine treatment, but upregulated with betahistine co-treatment. In addition, our epigenetic analysis showed that (1) betahistine co-treatment reversed the olanzapine-induced suppression of hepatic global H3K9me; (2) the mRNA expression of two H3K9me-specific histone demethylases (Kdm4b and Phf2) and a histone demethylase specific to H3K4 (Kdm1a) was significantly upregulated in the olanzapine-treated group, but downregulated in the O+B co-treatment group; (3) betahistine co-treatment improved the enrichment of both H3K9me binding on the *Pparg2* promoter and H3K4me binding on the *Cpt1a* promoter, restrained by the olanzapine treatment. These results suggest that betahistine co-treatment attenuated olanzapine-induced dyslipidemia and hepatic lipid accumulation through epigenetic histone modulation on key genes responding to hepatic lipid storage and fatty acid oxidation.

The upregulation of hepatic PPARγ is robustly related to fatty liver/steatosis in human and animal models [37,38]. Although single-nucleotide polymorphism studies have not identified an association between PPARγ and antipsychotic-induced weight gain in schizophrenia patients with olanzapine and clozapine treatment [39,40], our previous study indicated that the activation of the hepatic Pparγ pathway was linked to the olanzapine-induced hepatic adipogenesis and lipid accumulation in the liver [24]. A study in the 3T3-L1 cell model revealed that berberine alleviated olanzapine-induced adipogenesis by downregulating the expression of genes regulating the processes, including PPARγ [41]. This study revealed downregulated Pparγ expression in the betahistine co-treatment group compared to olanzapine-only treatment, accompanied by decreased plasma TG and NEFA concentrations, as well as reduced adiposity and lipid droplets in the liver. These results suggest that PPARγ signaling is involved in the effect of betahistine in preventing abnormal lipid metabolism caused by olanzapine. It is consistent with our previous report that betahistine-only treatment had no effects on lipid metabolism in the animal model [12,13]. C/EBPα is another critical regulator in the cascade of PPARγ pathway-mediated adipogenesis or lipid accumulation in the liver [42]. They have a broad overlap in their downstream transcriptional targets; moreover, they mutually stimulate each other and cross-regulate in maintaining the process of adipose differentiation [43]. In this study, C/ebpα expression was also found to be downregulated by olanzapine treatment, but upregulated by the O+B co-treatment. Moreover, a significant positive correlation in the protein concentration between Pparγ and C/ebpα was observed. CPT1A is a rate-limiting enzyme that facilitates fatty acid transport into the mitochondria for the subsequent process of oxidation, while CPT1A deficiency leads to fatty liver in humans (Bonnefont et al., 2004); this makes it an attractive target for therapeutic interventions [26]. A significant enhancement in *Cpt1a* mRNA and protein expression was observed in the O+B co-treatment. By contrast, Cpt1a was significantly inhibited by long-term olanzapine administration. Therefore, betahistine co-treatment may ameliorate olanzapine-induced lipid dysfunction, partly through promoting the CPT1A-mediated acceleration of fatty acid oxidation.

Our previous study showed that H3K9me, a silencing histone marker, played a crucial role in regulating the cascade of adipogenesis through modulating the promoter region of *PPARg* [24]. This study found that betahistine co-treatment significantly improved both the hepatic global levels of H3K9me and its specific binding on the promoter region of *Pparg* that were inhibited by chronic olanzapine treatment. PHF2 and KDM4B are the H3K9-specific histone demethylases and have been identified in modulating the lipid metabolism process [44,45]. Consistently, this study found that the mRNA expression of both *Phf2* and *Kdm4b* was increased by olanzapine-only treatment. This suggests that olanzapine promoted PHF2 and KDM4B to erase methylation on H3K9 and subsequently alleviate H3K9me binding on the promoter region of *PPARg*, leading to the acceleration of its expression and adipogenesis, whereas betahistine co-treatment decreased KDM4B expression. *Phf2* expression was also inhibited by the betahistine-only treatment, while there was also no significant difference in *Phf2* expression between the O+B and control groups. However, there were no differences in *Ehmt2* (a histone methyltransferase for H3K9me) between groups. Therefore, betahistine inhibits PHF2 and KDM4B to wipe off H3K9me, and then enhances H3K9me binding on the PPARγ2 promoter area and inhibits PPARγ expression and adipogenesis.

On the other hand, H3K4me, with the opposite effect to H3K9me, has been reported to be correlated with gene activation in metabolic pathways, including CPT1a-mediated fatty acid oxidation [34,35]. Similarly, this study found that betahistine significantly increased the global levels of H3K4me and its specific binding on the promoter region of *Cpt1a* in the liver. This provides the first evidence to suggest that H3K4me contributes to the activation of Cpt1a caused by betahistine. Meanwhile, KDM1A (LSD1) is a histone demethylase that erases methylation at K4 on histone H3 [46]. This study revealed the upregulation of *Kdm1a* mRNA expression in the olanzapine-only group and downregulation in the betahistine group compared to the control, while the co-treatment with betahistine reversed the increase in *Kdm1a* mRNA expression induced by olanzapine. However, there were no differences in *Kmt2c* (a histone methyltransferase for H3K4me) between groups. These results suggest that olanzapine promoted KDM1A, a histone demethylase, causing demethylation on H3K4, and subsequently reduced H3K4me binding on the CPT1A promoter region, which led to lower CPT1A expression and the process of fatty acid oxidation. On the other hand, betahistine co-treatment inhibits KDM1A to improve methylation on H3K4 and accelerates CPT1A expression and fatty acid oxidation. Interestingly, there were not any significant differences in H3K27me on both global levels and on the specific gene loci (both *Pparg* and *Cpt1a*), as well as no differences in *Ezh2* (a H3K27 methyltransferase) among the four groups. Although EZH2 has been previously reported to have a role in facilitating adipogenesis [47], results from this study suggest that H3K27me is not involved in the effects of olanzapine and/or betahistine treatment on adipogenesis.

It is worth noting that, in line with the finding of no clear enrichment of H3K4me on the Cpt1a promoter, there were not any significant changes in Cpt1a transcription following chronic olanzapine treatment. This outcome suggests that the reduction in Cpta1 protein levels induced by olanzapine may not be associated with transcriptional mechanisms, but translational or posttranslational ones. One limitation of this study is that only female rats have been investigated. The female model was chosen in this study because both preclinical and clinical studies reported that females showed more severe weight gain and metabolic responses to antipsychotic treatment, partly due to the effects of sex hormones such as estrogen [48,49]. Although there are sex differences, antipsychotics cause the accumulation of visceral adipose tissue and dyslipidemia in both male and female subjects [49,50]. Therefore, further studies are necessary to investigate whether betahistine has similar effects in preventing antipsychotic-induced dyslipidemia and other metabolic disorders in males. It should also be noted that the effects of betahistine on lipid metabolism in the rat model may not be directly relevant to the major effect of betahistine in clinical human studies, which requires further investigations.

In summary, this study further confirmed our previous report on the role of the histone modulation of the PPARγ pathway in olanzapine-induced metabolic disorders [24]. Importantly, this study provided novel evidence that betahistine co-treatment could enhance the enrichment of H3K4me and H3K9me binding on the promoter regions of *Cpt1a* and *Pparg*, respectively. This leads to an increase in the CPT1A pathway in promoting the progress of fatty acid oxidation but a decrease in the PPARγ pathway in inhibiting adipogenesis in the liver. Coupled with the inhibition effect of betahistine on the expression of *Kdm1a*, *Kdm4b*, and *Phf2*, this study demonstrated that the activation of the CPT1A pathway and the inhibition of the PPARγ pathway induced by co-treatment with betahistine were modulated by hepatic KDM1A-mediated H3K4me to *CPT1A*, and both KDM4B- and PHF2-mediated H3K9me to *PPARg*. Taken together, for the first time, this study reveals the epigenetic histone modulation mechanisms underlying the amelioration effect of betahistine co-treatment on dyslipidemia and hepatic adipose accumulation caused by olanzapine.

## 4. Materials and Methods

### 4.1. Animal Treatment

The animal experiment procedures were approved by the Animal Ethics Committee, University of Wollongong (AE11/10), and complied with the Australian Code of Practice for the Care and Use of Animals for Scientific Purposes (National Health and Medical Research Council, Australia, 2004). In order to examine the chronic effect of olanzapine and betahistine, the whole experimental period lasted for 11 weeks and was separated into three stages, as reported previously [13]. In brief, at stage one, after one week of environmental adaptation, 48 Sprague-Dawley female rats purchased from the Animal Resources Centre (Perth, WA, Australia) were randomly divided into two groups (*n* = 24/group) and treated with 3.5 weeks of cookie pellets (vehicle; 0.3 g, including 30.9% cornstarch, 30.9% sucrose, 6.3% gelatin, 15.5% casein, 6.4% fiber, 8.4% minerals, and 1.6% vitamins) or cookie pellets with olanzapine (1 mg/kg, 3 times/day; Eli Lilly, Indianapolis, IN, USA). At stage two, olanzapine was withdrawn for 2.5 weeks, during which none of the rats received any treatment. At stage three, the two groups were further divided into four subgroups (*n* = 12/subgroup) for further treatment for 5 weeks: (1) control (treated with cookie pellets without drug), (2) betahistine-only (9.6 mg/kg, 3 times/day; Manus Aktteva, Gujarat, India); (3) olanzapine-only (1 mg/kg, 3 times/day), (4) co-treated with olanzapine and betahistine (O+B; 1 mg/kg olanzapine plus 9.6 mg/kg betahistine, 3 times/day). The drug dosages were translated from human dosages to rats based on the body surface area and followed the FDA guidelines [51,52]. In consideration of the shorter half-lives of these drugs in rats than in humans, 1 mg/kg olanzapine and 9.6 mg/kg betahistine in rats are equivalent to ~10 mg olanzapine clinical dosage and ~48 mg betahistine used in clinical trials, respectively [10,14,16].

### 4.2. Plasma and Liver Lipid Assays

Plasma was separated from a 5 mL cardiac blood sample through centrifugation (3000 RPM for 15 min at 4 °C) immediately after the rats were euthanized by carbon dioxide asphyxiation. A Konelab 30i biochemistry analyzer (Thermo Fisher Scientific Oy, Vantaa, Finland) was used to measure total triglycerides (TG) and total cholesterol (TC). A non-esterified fatty acid (NEFA) ELISA kit (Wako Chemicals, Richmond, VA, USA) was used to test NEFA levels. Livers were dissected at 12 μm using a cryostat and stained with Oil Red O (Sigma–Aldrich 1516, St Louis, MO, USA) to evaluate hepatic lipid accumulation [13,16].

### 4.3. Examining Hepatic Gene Expression Using Quantitative Reverse Transcription PCR (qRT-PCR)

In brief, the Aurum ^TM^ RNA Mini Kit (Bio-Rad, Hercules, CA, USA) was used to extract the total hepatic RNA, and the iScript^TMRT^ Supermix Kit (Bio-Rad, Hercules, CA, USA) was used to synthesize the first chain of cDNA, according to the manufacturer’s manuals. The RNA was quantified using a Nano Drop 2000 (Thermo Fisher, Waltham, MA, USA). The 10.00 µL qRT-PCR reaction mixture, including 5.0µL TaqMan^®^Gene Expression Master Mix (Applied Biosystems™, Foster City, CA, USA), 0.50 µL TaqMan^®^Gene Expression Assay (Thermo Fisher, Waltham, MA, USA), 2.00 µL (5.00 ng/µL) cDNA, and 2.50 µL dH_2_O, was amplified on a Quant Studio 5 Real-Time PCR System (Thermo Fisher, Waltham, MA, USA), using the following parameters: 95 °C 10 min; 95 °C 15 s, 60 °C 1 min, 40 cycles. The nine target genes were *Pparg* (Rn00562597_m1), *Cebpα* (Rn00563565_m1), *Cpt1a* (Rn00580702_m1), *Kdm1a* (Rn01181029_m1), *Phf2* (Rn01435384_m1), *Kmt2*c (Rn01410347_m1), *Kdm4b* (Rn01527809_m1), *Ehmt2* (Rn01525918_m1), and *Ezh2* (Rn01500693_m1). The four endogenous control genes were *Actb* (Rn00667869 ml), *Rplp0* (Rn03302271_gH), *Hprt1* (Rn01527840_m1), and *Gapdh* (Rn01775763_gl). Each sample examination was performed twice. Endogenous control genes were used as the interior reference to normalize the original PCR data from target genes, and further relative expression was evaluated by the 2^−∆∆CT^ method.

### 4.4. Analyses of Global Histone Methylation Using Western Blot

To investigate whether histone methylation is involved in the modulation of the lipid metabolic signaling pathways, this study first evaluated the profiles of global histone methylation in H3K4me, H3K9me, and H3K27me. Protein from nearly 20 mg liver tissue was extracted using cell lysis buffer containing 9.80 mL NP40 (Invitrogen, Camarillo, CA, USA), 100 µL protease inhibitor cocktail (Sigma-Aldrich, St. Louis, MO, USA), 100 µL 50 mM β-Glycerophosphate (Invitrogen, Carlsbad, CA, USA), and 33.30 µL 0.3 M phenyl-methylsulfonyl fluoride (Sigma-Aldrich, St. Louis, MO, USA). The 8% to 12% sodium dodecyl sulfate–polyacrylamide gel and polyvinylidene difluoride membrane (Bio-Rad, Hercules, CA, USA) were, respectively, used in the electrophoretic separation and shifting of aliquots containing 10 μg of protein. After incubation with the first antibodies overnight at 4 °C and matching second antibodies for one hour at room temperature, images of the separated protein bands were achieved using the Amersham Hyperfilm Gel Imager (GE Healthcare, Life Science, FISHERS, IN, USA) and then quantified using a Fiji image processing package. The first antibodies were anti-H3K4me2 (1:1000, Abcam, ab176878; Cambridge, UK), anti-H3K9me2 (1:1000, Abcam, ab176882), anti-H3K27me2 (1:500, Abcam, ab194690), anti-*Cpt1a* (1:1000, Abcam, ab176320), anti-Pparγ (1:1000, Abcam, ab209350), anti-C/ebpα (1:1000, Abcam, ab40764), and anti-β-actin (1:3000, Merck, MAB1501; Darmstadt, Germany). The second antibodies were HRP-linked anti-rabbit IgG (1:3000, Cell Signaling, #7074s; Beverly, MA, USA) and HRP-linked anti-mouse IgG (1:3000, Cell Signaling, #7076s). All Western blot quantifications were normalized to the loading control and the untreated control values.

### 4.5. Analysis of DNA Fragment Binding on Histone Methylation Marks Using ChIP-qPCR

A chromatin immunoprecipitation (ChIP) experiment was applied to analyze DNA– protein interactions in the liver tissue using ChIP Kits for H3K4me2 (P-2009-48), H3K9me3 (P-2008-48), and H3K27me2 (P-2016-48; Epigentek, Farmingdale, NY, USA). Briefly, 40 mg liver tissue was homogenized by a Precellys^®^24 homogenizer (Bertin Technologies, Bretonneux, France). After completing the procedure of in vivo cross-linking with 1% formaldehyde, the suitable DNA fragment (200~1000 bp) was sheared using the Branson 450 Digital Sonifier (Branson, St. Louis, MO, USA) on ice. Strip microplate cups were used to perform the DNA–protein immunoprecipitation, while columns were used for ChIP DNA purification. Finally, quantitative PCRs for the special promoter region for *Pparg1* and *Pparg2*, as well as *Cpt1a*, were double analyzed using SYBR Green Master Mix (Qiagen, Germantown, MD, USA) on a Quant Studio 5 Real-Time PCR System (Thermo Fisher, Waltham, MA, USA), and qPCR data were shown as a % of input. The primers are referred to in the literature [35,53].

### 4.6. Statistical Analysis

SPSS 28 (IBM, Chicago, IL, USA) was used to analyze all data. The Kolmogorov–Smirnov test was used to examine the distribution of data from all experiments. All data were analyzed by two-way ANOVAs (betahistine × olanzapine), followed by a post-hoc Tukey test to perform multiple comparisons. If the data did not show a normal distribution, a nonparametric Mann–Whitney U test was used. Finally, the correlation among the measurements was analyzed by Pearson’s correlation test. Statistical significance was accepted when *p* < 0.05. Results were presented as the mean ± SEM. GraphPad Prism 7.04 (GraphPad Software Inc., San Diego, CA, USA) was used to create all graphs in this manuscript.

## Data Availability

The data sets used and analyzed in this study are available from the corresponding authors on request.

## Abbreviations

AMPK	activating protein kinase;
C/EBPs	CCAAT/enhancer binding proteins;
ChIP	chromatin immunoprecipitation;
CPT1A	carnitine palmitoyltransferase 1A;
FAS	fatty acid synthase;
H3K4me	histone H3 methylations at 4 lysine residue;
H3K9me	histone H3 methylations at 9 lysine residue;
KDM1A	lysine (K)-specific demethylase 1A;
KDM4B	lysine demethylase 4B;
NAFLD	non-alcoholic fatty liver disease;
NEFA	non-esterified fatty acid;
NPY	neuropeptide Y;
PPAR	peroxisome proliferator-activated receptor;
PHF2	PHD finger protein 2;
qRT-PCR	quantitative reverse transcription PCR;
SGAs	second-generation antipsychotics;
TC	cholesterol;
TG	total triglycerides.
